# Characteristics of Particles and Debris Released after Implantoplasty: A Comparative Study

**DOI:** 10.3390/ma15020602

**Published:** 2022-01-14

**Authors:** Xixi Wu, Changjie Cai, Javier Gil, Elizabeth Jantz, Yacoub Al Sakka, Miguel Padial-Molina, Fernando Suárez-López del Amo

**Affiliations:** 1 Department of Periodontics, College of Dentistry, University of Oklahoma, Oklahoma City, OK 73117, USA; xixi-wu@ouhsc.edu (X.W.); Elisabeth-jantz@ouhsc.edu (E.J.); 2 Department of Occupational and Environmental Health, Hudson College of Public Health, University of Oklahoma, Oklahoma City, OK 73117, USA; Changjie-cai@ouhsc.edu; 3 Bioengineering Institute of Technology, Universitat Internacional de Catalunya, Josep Trueta s/n. Sant Cugat del Vallés, 08125 Barcelona, Spain; 4 Facultat de Odontologia, Universitat Internacional de Catalunya, Josep Trueta s/n. San Cugat del Vallés, 08125 Barcelona, Spain; 5 Private Practice, Edmond, OK 73013, USA; y.sakka@uok.edu; 6 Department of Oral Surgery and Implant Dentistry, School of Dentistry, University of Granada, 18071 Granada, Spain; mipadial@ugr.es; 7 Private Practice, 28050 Madrid, Spain

**Keywords:** implantoplasty, titanium particles, debris, nano particles, dental implant, peri-implantitis, titanium alloy

## Abstract

Titanium particles embedded on peri-implant tissues are associated with a variety of detrimental effects. Given that the characteristics of these detached fragments (size, concentration, etc.) dictate the potential cytotoxicity and biological repercussions exerted, it is of paramount importance to investigate the properties of these debris. This study compares the characteristics of particles released among different implant systems (Group A: Straumann, Group B: BioHorizons and Group C: Zimmer) during implantoplasty. A novel experimental system was utilized for measuring and collecting particles generated from implantoplasty. A scanning mobility particle sizer, aerodynamic particle sizer, nano micro-orifice uniform deposit impactor, and scanning electron microscope were used to collect and analyze the particles by size. The chemical composition of the particles was analyzed by highly sensitive microanalysis, microstructures by scanning electron microscope and the mechanical properties by nanoindentation equipment. Particles released by implantoplasty showed bimodal size distributions, with the majority of particles in the ultrafine size range (<100 nm) for all groups. Statistical analysis indicated a significant difference among all implant systems in terms of the particle number size distribution (*p* < 0.0001), with the highest concentration in Group B and lowest in Group C, in both fine and ultrafine modes. Significant differences among all groups (*p* < 0.0001) were also observed for the other two metrics, with the highest concentration of particle mass and surface area in Group B and lowest in Group C, in both fine and ultrafine modes. For coarse particles (>1 µm), no significant difference was detected among groups in terms of particle number or mass, but a significantly smaller surface area was found in Group A as compared to Group B (*p* = 0.02) and Group C (*p* = 0.005). The 1 first minute of procedures had a higher number concentration compared to the second and third minutes. SEM-EDS analysis showed different morphologies for various implant systems. These results can be explained by the differences in the chemical composition and microstructures of the different dental implants. Group B is softer than Groups A and C due to the laser treatment in the neck producing an increase of the grain size. The hardest implants were those of Group C due to the cold-strained titanium alloy, and consequently they displayed lower release than Groups A and B. Implantoplasty was associated with debris particle release, with the majority of particles at nanometric dimensions. BioHorizons implants released more particles compared to Straumann and Zimmer. Due to the widespread use of implantoplasty, it is of key importance to understand the characteristics of the generated debris. This is the first study to detect, quantify and analyze the debris/particles released from dental implants during implantoplasty including the full range of particle sizes, including both micro- and nano-scales.

## 1. Introduction

Titanium and its alloys perform excellently for implant osseointegration, and therefore have seen widespread use as biomaterials in medical and dental fields [1]. Titanium dental implants have favorable properties, such as low specific weight, high corrosion resistance, and excellent general biocompatibility [2]. However, dental implants are not exempt from complications. Beside the extensively studied inflammatory peri-implant diseases (i.e., mucositis and peri-implantitis), some investigations have reported hypersensitivity and allergic reactions to titanium [3], increased titanium concentration in serum [4] and urine [5] and the presence of particles/ions adjacent to dental implants and at distant locations (lymph nodes, lungs, liver etc.) [6,7,8].

Peri-implantitis, characterized by inflammation of the peri-implant mucosa and subsequent progressive loss of supporting bone, is defined as a plaque-associated pathological condition [9] with multiple local-, systemic-, implant- and host-related factors playing a role in its development and progression. Factors such as poor oral hygiene, history of periodontitis and smoking, among others, have been widely studied in relation to peri-implantitis. More recently, titanium particles and ions have also been investigated as potential etiologic and/or contributory factors for peri-implantitis [10,11,12]. Indeed, there are several studies linking the presence of titanium particles to peri-implantitis [12,13]. These metal debris have been associated with a variety of detrimental effects, including the initiation of an inflammatory process potentially leading to marginal bone loss [14,15], activation of DNA damage in oral epithelial cells [16] and ultimately peri-implantitis [12,13,17].

It has been demonstrated that particulate debris, acting as a biologically active substance, may initiate an osteolytic reaction leading to the failure of joint replacements [18]. Moreover, different compositions, concentrations, morphologies and sizes of metal particles exert different cytotoxic effects [19]. Among these factors, the size and concentration of the particles have a greater impact [18,20]. Higher levels of biological and immunological activity to metal particles have been related within the size range of 1–50 µm, and/or at the nano-level [6,7,8,12,14,21]. Most wear particles found in failed joint replacements are submicron (<1 µm) in size, and are more likely to be responsible for the osteolytic response initiated and mediated by macrophages [15,18,20]. Specifically, macrophages can phagocytose wear debris [22], resulting in the production of pro-inflammatory signal molecules such as prostaglandin E2 (PGE2), tumor necrosis factor alpha (TNF-α), interleukin-1β (IL1-β) and interleukin 6 (IL-6) [23]. These cytokines have been proved to be upregulated when the particle size falls within a certain range (0.6–4.5 µm) [24].

To promote successful osseointegration and faster bone healing, implant manufacturers have incorporated various surface treatments, including blasting, acid etching, plasma spraying, anodization etc. [25]. Nevertheless, these modifications may facilitate the release of wear particles during implant manipulation, such as placement, maintenance, peri-implant disease management etc. This alteration of the implant surface can be affected by the structure of the fixture, roughness and topographical configuration [26], and could influence the progression of peri-implantitis and/or its treatment [27].

Implantoplasty has been used for the surgical treatment of peri-implantitis, with some investigations reporting stable outcomes over time [28], although more recent evidence has demonstrated no additional benefits compared to less-aggressive modalities for the treatment of the implant surface [29]. Nevertheless, due to the widespread use of implantoplasty, it is of key importance to understand the characteristics of the generated debris. Therefore, this study aimed at investigating the debris particles generated with implantoplasty, including particles’ size, number concentration, mass, surface area and morphology. Additionally, the differences among different implant manufacturers were also evaluated.

## 2. Materials and Methods

### 2.1. Experimental System

**Figure 1:**
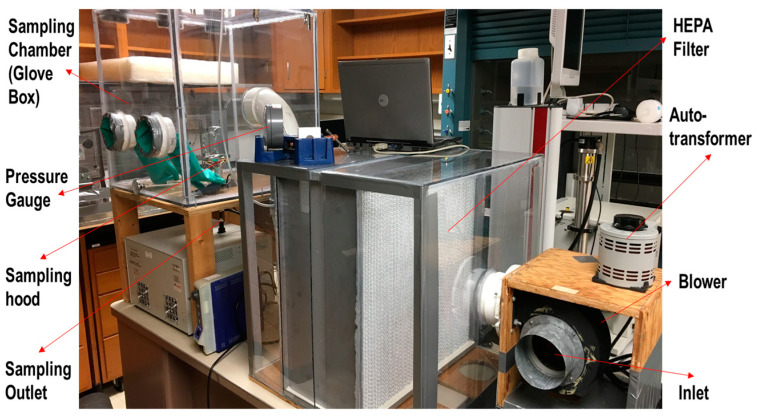
Experimental system employed for collecting and quantifying particles.

A novel experimental system was created and utilized for collecting and quantifying particles generated from implantoplasty (Figure 1).

A blower controlled by an autotransformer blew room air through one high-efficiency particulate air filter (HEPA) into the sampling chamber. A scanning mobility particle sizer (SMPS 3936, TSI Inc., Shoreview, MN, USA), aerodynamic particle sizer (APS 3314, TSI Inc., Shoreview, MN, USA) and nano micro-orifice uniform deposit impactor (0.2 µm pore size, 47 mm in diameter, Sterlitech Corporation, Kent, WA, USA) were installed outside the system with sealed tubes connecting to the chamber. Mixed cellulose esters (MCE) filters (0.8 µm pore size, 47 mm diameter, Zefon International, Inc., Ocala, FL, USA) were inserted in the nanoMOUDI for particle collection.

### 2.2. Implants

Nine implants from three implant systems were tested (Table 1):

- Group A (Straumann^®^ BLT, RC, SLA; 4.1 Ø mm, length 16 mm; Institut Straumann AG, Basel, Switzerland);
- Group B (BioHorizons^®^ Tapered Plus; 4.6 mm × 15 mm; BioHorizons Plus, Birmingham, AL, USA).
- Group C (Zimmer^®^ Tapered Screw-Vent MTX; 4.1 mm × 16 mm; Zimmer Dental Inc., Carlsbad, CA, USA).

**Figure 2:**
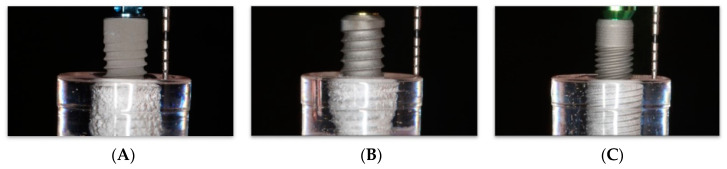
Implants embedded in epoxy resin with 6 mm of surface exposed. (**A**) Straumann; (**B**) BioHorizons; (**C**) Zimmer.

All implants were embedded in epoxy resin (crystal clear; vinyl cyclohexene dioxide; East Coast^®^) contained in Falcon round-bottom polystyrene test tubes (5 mL, Polystyrene; Corning^®^) (Figure 2). Implant recipient sites were drilled in each resin tube using a standard surgical drill sequence with ceramic sequential drills (Komet, Gebr. Brasseler GmbH & Co., KG, Lemgo, Germany) including round bur, 2.2 mm, 2.8 mm and 3.5 mm drills. Later, implants were inserted, leaving the most coronal 6 mm exposed, resembling a circumferential peri-implant defect [28].

### 2.3. Intra-Operator Calibration

Before the experiment, the operator repeatedly performed implantoplasty for three implants and results were analyzed with SMPS and APS data for calibration. In the aerosol research field, particles are commonly categorized into three modes: an ultrafine mode (<0.1 µm), a fine mode (0.1~1 µm) and a coarse mode (>1 µm). Data from these procedures showed bimodal curves with the median of each mode (coarse, fine, ultrafine) with <10% variation, indicating adequate intra-operator consistency.

### 2.4. Implantoplasty Procedures

The implants were subjected to the implantoplasty protocol using rotary tungsten carbide round burs (Brasseler USA, Savannah, GA, USA). One calibrated operator (XW) performed all implantoplasty procedures under standardized conditions. Three tested implants per implant system were included to ensure data consistency. A hand-held straight hand-piece (at 40,000 rotations per min/rpm) was used to perform the procedure until all implants had an evenly machined appearance to the naked eye. The duration of the procedure was 3 min per implant.

### 2.5. Particle Collection and Analysis

SMPS was used for the analysis of particle number concentration by size from ~10 nm to ~700 nm, and APS was used to measure particle number concentration by size from ~700 nm to ~20 µm. Particles in this size range (~10 nm to ~20 µm) are believed to cover most implant debris, according to previous studies [6,7,8,12,14,21,30]. In this study, the data of particle number, mass and surface area concentration were collected from SMPS and APS and subsequently analyzed. In addition, nanoMOUDI was utilized to collect particles by size onto 13 substrates arranged sequentially, collecting particles with difference size range/diameter ranging from >~10 μm (level 2) to <~0.01 μm (level 15). The diameter used in nanoMOUDI is the aerodynamic diameter, which has two assumptions: (1) unit density (water density), (2) spherical shape [31]. In the current investigations, we assumed that particles met these two requirements. Last, scanning electron microscopy with a backscattered electron detector (SEM-BSD) and X-ray microanalysis were performed in order to determine the chemical composition, with a high-sensitivity beryllium detector (Jeol 6400, Tokyo, Japan). This analysis was carried out to determine the composition and morphology of the collected particles by size. The released debris were characterized by scanning electron microscopy (SEM) using a Phenom XL Desktop SEM microscope (PhenomWorld, Eindhoven, The Netherland) with a voltage of 20 keV. In order to increase the conductivity of the debris, they were attached a high-purity carbon, improving the images obtained [32].

### 2.6. Mechanical Properties

Nanoindentation was used to determine the mechanical properties (maximum strength, 0.2% yield stress, elastic modulus, ductility and hardness) of the released particles during implantoplasty. Tests were realized using a “Berkovich” indenter, with a constant strain rate of 0.05 s^−1^. The equipment used were an iMiro (KLA-Tencor) and a Nanoindenter XP (MTS Systems Corporation, Oak Ridge, MN, USA). The nanoindentation equipment used exerts a charge on the surface of the growing nanoparticle that penetrates inwards. The system can determine the depth and can be assimilated to deformation. In other words, nanoindentation works like a compression test. The mechanical equipment was coupled to a high-resolution microscope to determine where the indentation was made. Fifteen nanoindentation tests were performed in order to have a representative sample.

### 2.7. Statistical Analysis

Non-parametric Kruskal–Wallis was conducted to compare the median measures of the particles released among the three implant systems. The level of statistical significance was set to 0.05 for this study. Multiple pairwise comparisons using the DSCF method informs of the relative impact of the implant manufacturer on each outcome. The analysis for each outcome is stratified by duration (1, 2, 3 min) and categorical classification of particle size (coarse, fine, ultrafine).

## 3. Results

**Figure 3:**
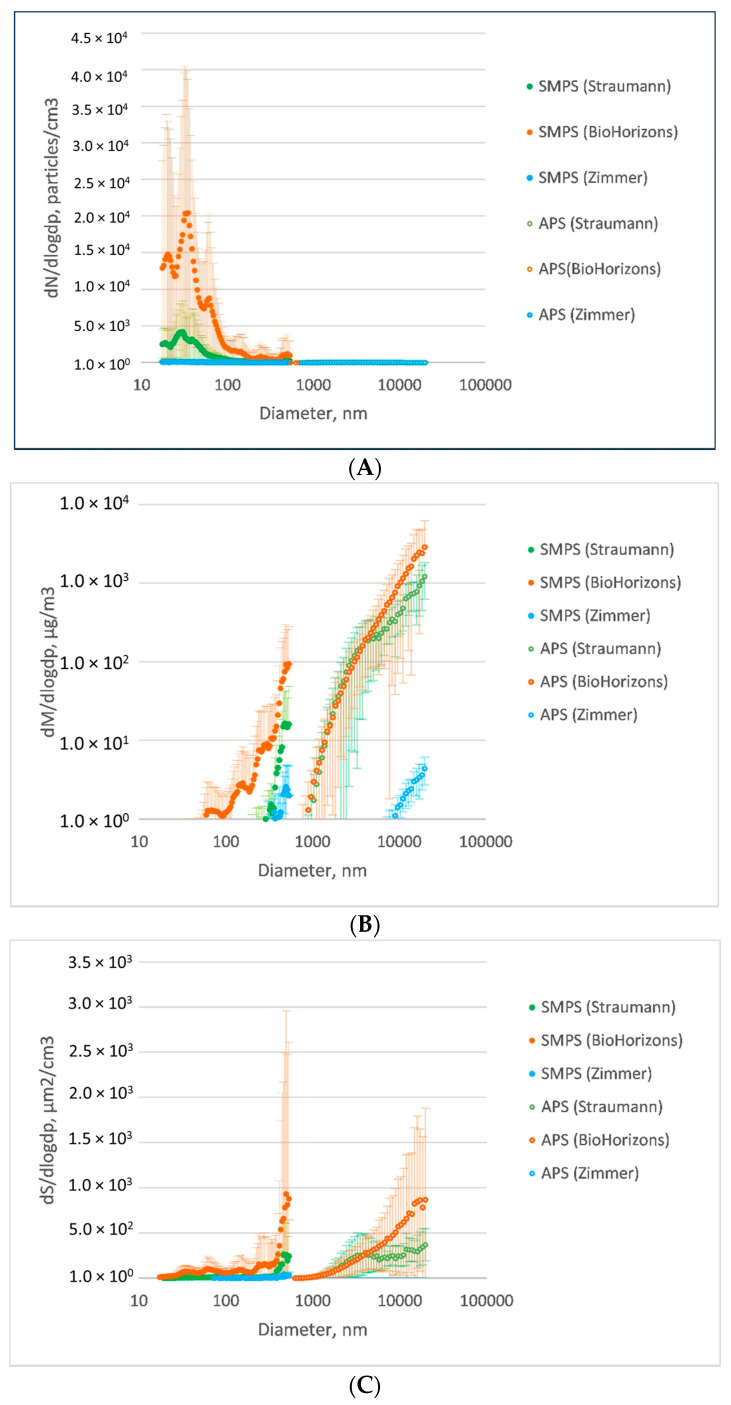
(**A**) Particle number concentrations distributed by size among groups. The highest particle number concentration was observed in Group B (BioHorizons); the lowest particle number concentration was in Group C (Zimmer), in both fine and ultrafine modes (<1 µm). For coarse particles (>1 µm), the particle number concentrations were more overlapped among groups. (**B**) Particle mass concentrations distributed by size among groups. The highest concentration of particle mass was observed in Group B (BioHorizons) and the lowest in Group C (Zimmer), in both fine and ultrafine modes (<1 µm). For coarse particles (>1 µm), Group A (Straumann) and Group B (BioHorizons) showed higher concentrations than Group C (Zimmer). (**C**) Particle surface area distributed by size among groups. The highest concentration of particle surface area was shown in Group B (BioHorizons) and the lowest in Group C (Zimmer), in both fine and ultrafine modes (<1 µm). For coarse particles (>1 µm), the difference was less significant. Green dots: Group A (Straumann); orange dots: Group B (BioHorizons); blue dots: Group C (Zimmer). Solid dots: data from SMPS; hollow dots: data from APS.

**Figure 4:**
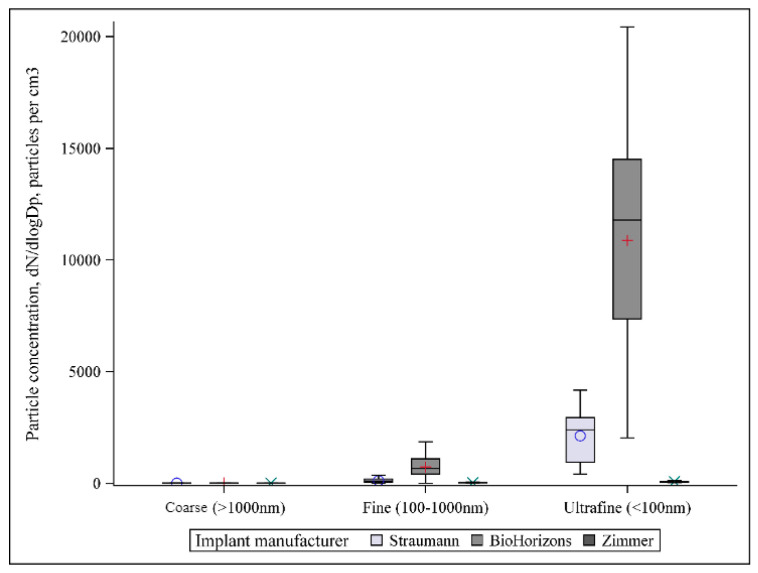
The mean particle number concentration from SMPS & APS stratified by particle size.

Particles emitted from the implant surface during implantoplasty showed bimodal number size distributions, with the majority of particles in the ultrafine size range (<100 nm) (Figure 3A). Most differences among groups were within fine (100–1000 nm) and ultrafine (<100 nm) particle size ranges (Figure 4). For particle number concentration, statistical analysis indicated significant differences among all manufacturers in terms of particle size distribution (*p* < 0.0001), with the highest number concentration in Group B (BioHorizons) and the lowest in Group C (Zimmer), in both fine and ultrafine modes (Table 2, Figure 3B). Significant differences among all groups (*p* < 0.0001) were also observed for the two other metrics, with the highest concentration of particle mass and surface area in Group B (BioHorizons) and the lowest in Group C (Zimmer), in both fine and ultrafine modes (Table 2, Figure 3B,C). For coarse particles (>1 µm), no significant differences were detected among groups in terms of particle number and mass, but a significantly smaller surface area was found in Group A (Straumann) as compared to Group B (*p* = 0.02) and Group C (*p* = 0.005) (Table 2, Figure 3A–C). 

**Figure 5:**
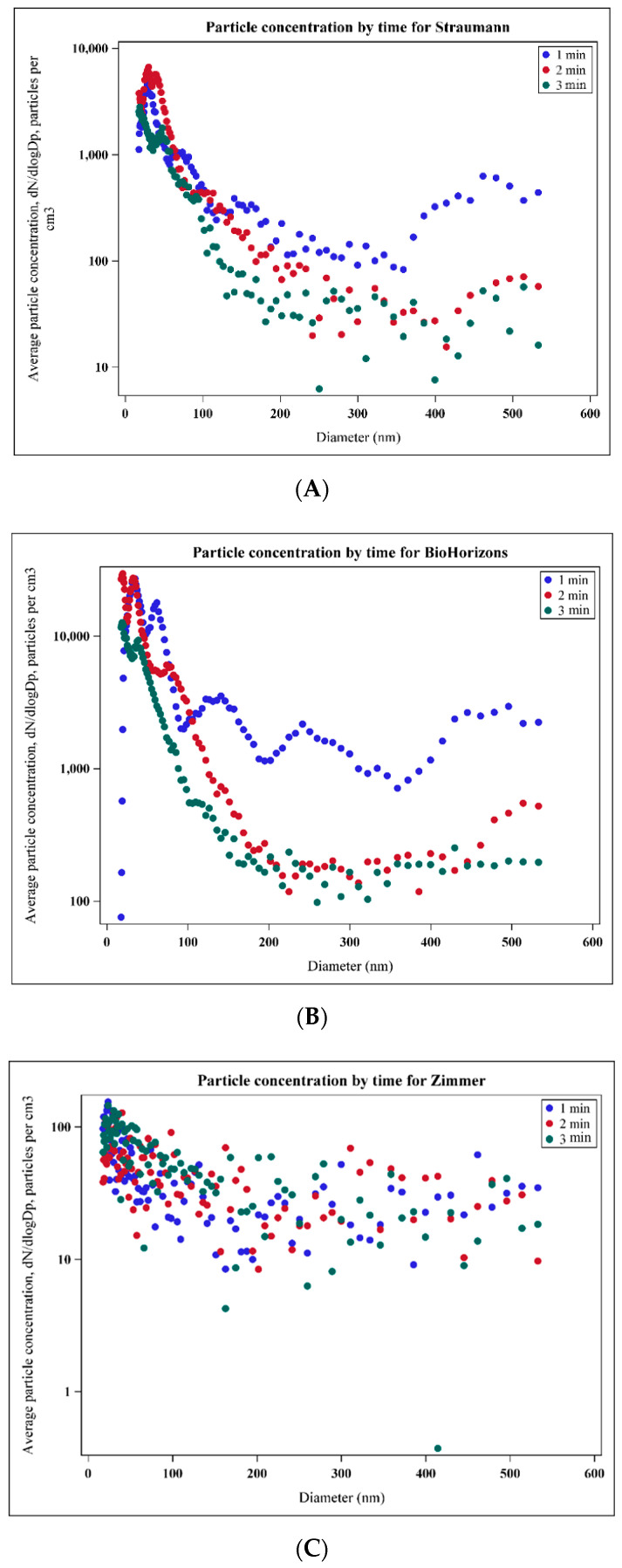
Dot plots indicating mean particle number concentration stratified by minute for (**A**) Straumann; (**B**) BioHorizons; (**C**) Zimmer.

Dot plots were created to indicate mean particle number concentration stratified by minute. The first minute of the procedure generated a higher number concentration compared to the second and third minutes for all implant systems, especially for Group A (Straumann) and Group B (BioHorizons) (Figure 5A–C).

**Figure 6:**
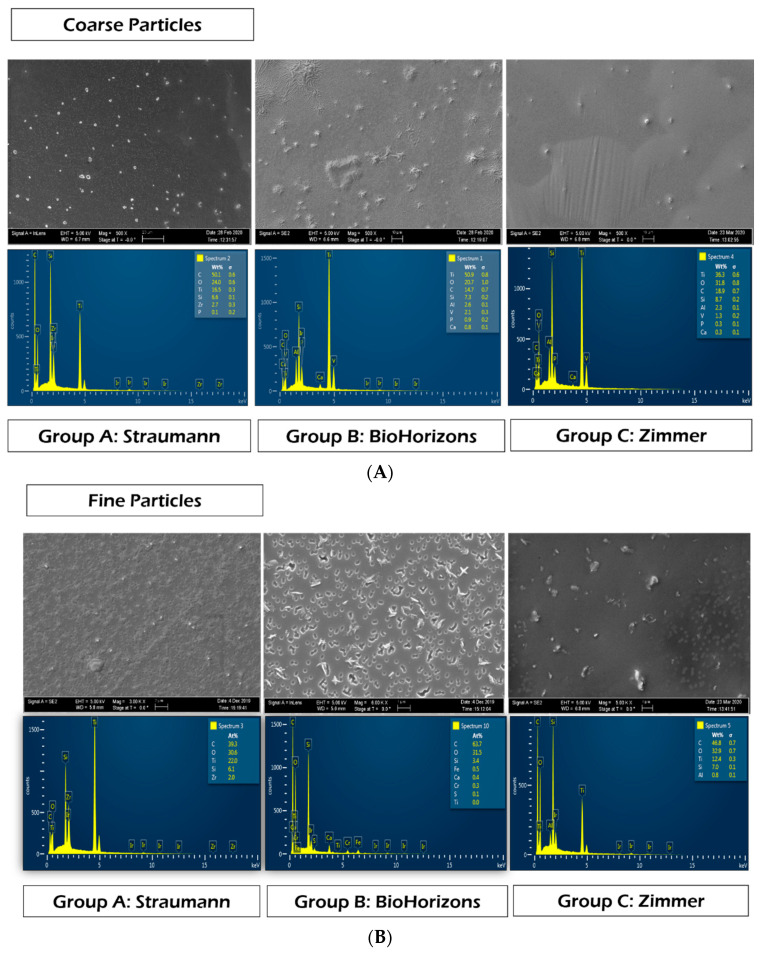

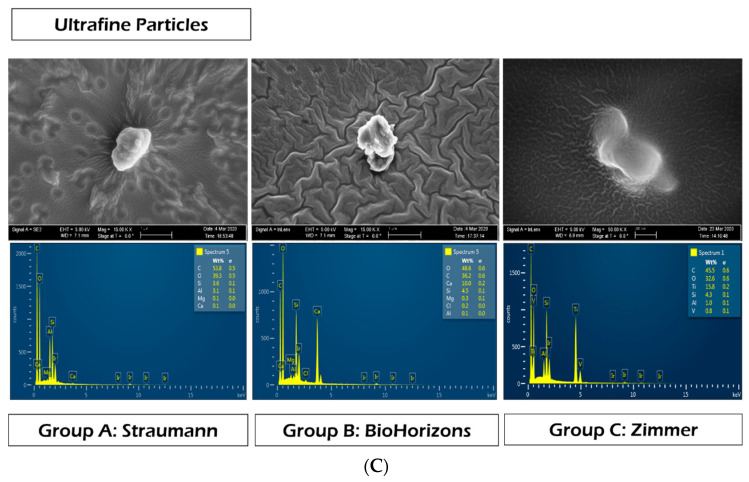
SEM-EDS analysis for (**A**) coarse particles; (**B**) fine particles; (**C**) ultrafine particles.

Finally, SEM-EDS analysis of nanoMOUDI-collected particles indicated that for the various implant systems, particles showed different morphology and composition. BioHorizons implants produced particles with more spindle/shaving shape (Figure 6) while particles from the other two groups seemed more spherical (Figure 6). In most samples, the elements Ti, C, O, Si and Al were detected. Interestingly, a higher percentage of titanium was detected by EDS within the coarse size range compared to fine and ultrafine particles (Figure 6). Moreover, titanium was not identified in ultrafine particles in either Group B (BioHorizons) or Group C (Zimmer) (Figure 6). Zr was detected only in Group A (Straumann). The specific percentage of elements is not presented due to the inaccuracy of element demonstration by EDS.

The compressive properties of the tested implants are shown in Table 3. From the results it can be seen that the Ti15Zr alloy had the highest hardness values and the Ti6Al4V alloy used in Zimmer dental implants had the highest mechanical strength values. The BioHorizons dental implants, which have the same alloy as Zimmer, had significantly lower values than the Straumann and Zimmer implants due to the laser treatment to which the dental implants are subjected. 

**Figure 7:**
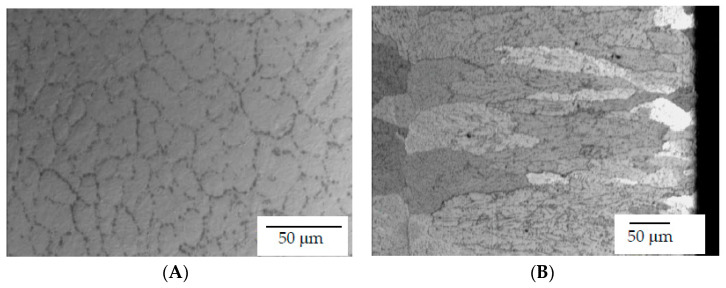
(**A**) Microstructure of the Ti6Al4V of a Zimmer dental implant. (**B**) Microstructure of the Ti6Al4V of a BioHorizons dental implant with laser treatment.

The BioHorizons Laser-Lok^®^ is a series of precision-engineered cell-sized channels laser-machined onto the surface of the dental implants and abutments. The surface treatment is intended to attract a true, physical connective tissue attachment. The treatment of laser produced an increase of the grain size, as can be observed in Figure 7. Figure 7A shows the microstructure of the Ti6Al4V of the Zimmer implant, with an average grain diameter of around 11 μm; Figure 7B shows the BioHorizons implant, with an average grain diameter of around 240 μm with a columnar shape. This larger gran size is due to the laser treatment because the high temperature produces grain boundary diffusion [33,34,35,36].

## 4. Discussion

This in vitro study demonstrated that performing implantoplasty on dental implants produces debris mostly within the ultrafine size range (<100 nm). Evidence from previous in vitro investigations have also indicated that nanometer-sized particles may account for the greatest number of debris generated, but only accounting for a very small proportion of the total volume [19,37]. These results indicate that a surgical area that seems visually “free of particles” after implantoplasty might still contain a great number of nanometric debris. Of importance, as reported previously in the orthopedic literature [38,39], the smaller particles (sub-micrometer-sized) have been shown to be more associated with macrophage activities, including increased cytokine release (e.g., TNF-α IL-1 β IL-6, IL-8, IL-11, TGF-β. Consequently, results from this investigation demonstrate that most of the particles released after implantoplasty were within the range of debris capable of exerting high levels of biological and immunological activity. These ultrafine debris are biologically more detrimental compared to the visible coarse particles, through biochemical mediators of inflammation, cellular recruitment and bone resorption [15]. As such, clinicians should consider alternatives to reduce the widespread release of particles generated during implantoplasty (rubber dam, high-volume evacuation, etc.). Similarly, the risks and benefits of performing implantoplasty for the treatment of the implant surface should be cautiously weighed for every implant/patient.

To the best of our knowledge, this is the first study to detect, quantify and analyze debris/particles released from dental implants during implantoplasty including size ranges from both micro- and nano-scales. Numerous studies have previously shown the presence of titanium particles in peri-implant mucosa [6,12], especially around fixtures suffering from peri-implantitis [13,21,30,40,41,42], but these investigations failed to perform a detailed description and analysis and the metal debris investigated. Most importantly, previous studies were not able to quantify the particles embedded on the peri-implant tissues. Given the paramount importance of the particles’ characteristics (i.e., size, concentration, composition, morphology), this study focused on the description of these factors by reporting number, size, mass and surface area. Results from this study can lead to future investigation evaluating the deleterious effects of these nano-scale titanium particles on peri-implant tissues, taking as a reference the debris observed in this study. 

This in vitro study also demonstrated differences in terms of number, size, mass, surface area and morphology among various manufacturers with different implant design and surface treatment. Nevertheless, the diffractograms of all samples showed similar bimodal curves. On the other hand, the generated particles showed differences regarding the concentration of number, mass and surface area. In this study, not only particle numbers were reported, but also particle mass and surface; this is because mass concentration is often not sufficient to fully describe small particles, whereas other metrics such as particle number or surface area may be more descriptive for ultrafine and fine particles [40]. Similarly, results also demonstrated that most particles were released within the first minute of implantoplasty, and the particles number concentration was reduced when reaching the second and third minutes, especially for Groups A and B. 

Within the coarse size range, elements such as C, O, Ti, Si and Al were found in all groups, which is consistent with previous investigations [12]. Zr was only observed in group A. For particles within fine and ultrafine size ranges, similar elements were observed. Interestingly, for these levels, the concentration of Ti decreased while those of C and O increased. Ti was only detected in Group C (Zimmer) in ultrafine particles. Results failed to detect Ti in ultrafine particles in the other two groups, probably due to the limitation of element detection by SEM/EDS analysis, in which spectrum point analysis was used. Therefore, it would be ideal to utilize spectrometry for composition analysis in future investigations.

It is important to bear in mind that a force-controlled rotary machine was considered for the current investigation. Nevertheless, an operator-controlled design was utilized in order to more closely resemble the clinical treatment of peri-implantitis. In addition, to avoid operation errors, intra-operator calibration was performed.

The mechanical properties analysis showed that the laser treatment applied to BioHorizons dental implants to improve the biological seal caused an increase in the grain size of the titanium alloy. According to the Hall–Petch relationship, this increase in grain size causes a decrease in the hardness values, causing the material to fracture more easily. Therefore, the particles were more numerous during implantoplasty. Zimmer’s Ti6Al4V alloy had smaller grain sizes and therefore had higher hardness and greater mechanical strength, as we were able to verify in the results of the mechanical tests. For this reason, the released particles were smaller in number [34,35,36].

The Straumann Ti15Zr alloy had higher hardness values but lower mechanical strength, which resulted in a slightly higher particle release in comparison to Zimmer, but a significantly lower release than BioHorizons.

Limitations of the current investigation include the utilization of implantoplasty as the only method for implant surface decontamination/modification. Often, implantoplasty is used in combination with other chemical and/or mechanical methods for implant detoxification

The influence of the particles should be studied, especially on the reaction of inflammatory cells, and to determine the activation of the immune system by nanometric particles. It is also important to determine the influence of the particles not only on peri-implantitis but also on other infections or the relationship between them. Gherlone et al. [43] conducted a comprehensive clinical study of dental implant placement including patients with HIV and observed a high incidence of peri-implant infections in the first six months.

The effect of granulocyte-macrophage colony-stimulating factor (GM-CSF) administration in oral cavity carcinoma patients should also be considered. The administration of 5 mg on the same day as radiotherapy significantly reduced mucositis in [44]. This research could help to determine a way to cure or at least reduce peri-implantitis. 

Peri-implantitis is an infection in which oral hygiene undoubtedly plays a role in its generation, but sometimes people with very good oral hygiene can be observed with infection, or vice versa, people with poor oral hygiene can be observed without infection. Therefore, the generation of infection can have many variables to consider: genetic factors, hygiene, the presence of particles that facilitate infection or the distribution of occlusal contacts that can cause disorders in the oral cavity [17,38,39,40,42,45].

Therefore, future testing is warranted to evaluate the occurrence of this phenomenon using different techniques, and within the oral environment. This study is part of a series of testing conditions that will investigate the debris particles released under various mechanical and electrochemical conditions from different implants with various materials and surface treatments. To further elaborate on this topic, further studies are needed to investigate the biotoxicity of the different types of debris generated during implantoplasty and the potential detrimental effects on clinical outcomes. Future studies should also evaluate the effects of these particles on cell behavior and utilize animal models to investigate cytotoxicity based on different particle size ranges, especially at the nano-scale.

## 5. Conclusions

Implantoplasty is associated with debris particle release, with the majority of particles being within the ultrafine size range (<100 nm). BioHorizons implants released more particles compared to Straumann and Zimmer systems. The first minute of the procedure released the most debris in terms of the particle number. The laser treatment of BioHorizons dental implants to improve the biological seal causes a decrease in the implant’s mechanical properties due to an increase in grain size, which explains the increased release of particles. The potential short- and long-term cytotoxicity and biological effects of these debris should be further investigated.

## Figures and Tables

**Table 1:** Comparison between different implant systems.

Manufacturer	Material	Current Generation	Technique	Chemical Treatment	Nanoscale
Straumann, Switzerland	Ti-15Zr	SLA	Blasting and acid-etched	N	N
Zimmer, Warsaw, IN, USA	Ti-6Al-4V	MTX	Blasting	N	N
BioHorizons, Birmingham, AL, USA	Ti-6Al-4V	Laser-Lok	Blasting	N	N

**Table 2:** Size distribution comparison among implant manufacturers stratified by particle size (particle number size; particle mass; particle surface area).

		Comparisons	Pairwise Comparisons ^b^		
	Particle Size	*p*-Value ^a^	Straumann vs. BioHorizons	Straumann vs. Zimmer	BioHorizons vs. Zimmer
**Particle number size**	Coarse (>1000 nm)	0.7169	<0.0001	-	-
	Fine (100–1000 nm)	<0.0001	<0.0001	<0.0001	<0.0001
	Ultrafine (<100 nm)	<0.0001	<0.0001	<0.0001	<0.0001
**Particle mass**	Coarse (>1000 nm)	0.0488	<0.0001	-	-
	Fine (100–1000 nm)	<0.0001	<0.0001	<0.0001	<0.0001
	Ultrafine (<100 nm)	<0.0001	<0.0001	<0.0001	<0.0001
**Particle surface area**	Coarse (>1000 nm)	0.0009	0.0232	0.0050	0.0547
	Fine (100–1000 nm)	<0.0001	<0.0001	<0.0001	<0.0001
	Ultrafine (<100 nm)	<0.0001	<0.0001	<0.0001	<0.0001

^a^ Kruskal–Wallis test for comparing median measurements. ^b^ Pairwise comparisons computed with DSCF method. If the overall *p*-value was >0.001 then the pairwise *p*-values are not displayed.

**Table 3:** Compressive properties of the debris studied. The standard deviation is given in brackets.

Implant System	Implant	Maximum Strength (MPa)	Yield Stress 0.2% (MPa)	Ductility (%)	Hardness (GPa)
Straumann	Ti15Zr	897 (24)	698 (20)	22 (4)	1.952 (137)
BioHorizons	Ti6Al4V *	860 (37)	657 (23)	17 (4)	1.118 (198)
Zimmer	Ti6Al4V	1050 (35)	740 (23)	8 (2)	1.451 (233)

* Laser treatment.

## Data Availability

The datasets generated during and/or analysed during the current study are available from the corresponding author on reasonable request.
